# PEG-Coated Large Mesoporous Silicas as Smart Platform for Protein Delivery and Their Use in a Collagen-Based Formulation for 3D Printing

**DOI:** 10.3390/ijms22041718

**Published:** 2021-02-09

**Authors:** Federica Banche-Niclot, Giorgia Montalbano, Sonia Fiorilli, Chiara Vitale-Brovarone

**Affiliations:** 1 Department of Applied Science and Technology, Politecnico di Torino, 10029 Torino, Italy; federica.banche@polito.it (F.B.-N.); giorgia.montalbano@polito.it (G.M.); sonia.fiorilli@polito.it (S.F.); 2 Department of Surgical Science, Università degli Studi di Torino, 10029 Torino, Italy; 3 National Interuniversity Consortium of Materials Science and Technology (RU Politecnico di Torino), 50121 Firenze, Italy

**Keywords:** mesoporous silica particles, large pores, growth factor, pH-triggered release, type I collagen, 3D printing, hydrothermal treatment

## Abstract

Silica-based mesoporous systems have gained great interest in drug delivery applications due to their excellent biocompatibility and high loading capability. However, these materials face challenges in terms of pore-size limitations since they are characterized by nanopores ranging between 6–8 nm and thus unsuitable to host large molecular weight molecules such as proteins, enzymes and growth factors (GFs). In this work, for an application in the field of bone regeneration, large-pore mesoporous silicas (LPMSs) were developed to vehicle large biomolecules and release them under a pH stimulus. Considering bone remodeling, the proposed pH-triggered mechanism aims to mimic the release of GFs encased in the bone matrix due to bone resorption by osteoclasts (OCs) and the associated pH drop. To this aim, LPMSs were prepared by using 1,3,5-trimethyl benzene (TMB) as a swelling agent and the synthesis solution was hydrothermally treated and the influence of different process temperatures and durations on the resulting mesostructure was investigated. The synthesized particles exhibited a cage-like mesoporous structure with accessible pores of diameter up to 23 nm. LPMSs produced at 140 °C for 24 h showed the best compromise in terms of specific surface area, pores size and shape and hence, were selected for further experiments. Horseradish peroxidase (HRP) was used as model protein to evaluate the ability of the LPMSs to adsorb and release large biomolecules. After HRP-loading, LPMSs were coated with a pH-responsive polymer, poly(ethylene glycol) (PEG), allowing the release of the incorporated biomolecules in response to a pH decrease, in an attempt to mimic GFs release in bone under the acidic pH generated by the resorption activity of OCs. The reported results proved that PEG-coated carriers released HRP more quickly in an acidic environment, due to the protonation of PEG at low pH that catalyzes polymer hydrolysis reaction. Our findings indicate that LPMSs could be used as carriers to deliver large biomolecules and prove the effectiveness of PEG as pH-responsive coating. Finally, as proof of concept, a collagen-based suspension was obtained by incorporating PEG-coated LPMS carriers into a type I collagen matrix with the aim of designing a hybrid formulation for 3D-printing of bone scaffolds.

## 1. Introduction

Drug delivery systems (DDSs) are designed to control the release of therapeutic substances with the aim of imparting a specific effect in humans or animals [1,2] and have been widely used to improve dosing efficiency and safety by controlling the kinetics and the site of drug release in the body [1,3]. These systems include nano- and microparticles, which must be biocompatible, non-toxic as well as safe and, hence, if accumulated in the body for a certain period, they must not cause undesirable effects [2,4].

Over the last two decades, the research attention on DDSs based on inorganic materials like mesoporous silica particles, gold nanoparticles, and graphene oxide [5] has seen rapid growth in the field of biomedicine, expanding in many directions. These materials were employed as carriers of anti-inflammatory agents [6,7,8], tumor diagnostic probes [9,10], as well as in biomedical imaging [11,12] and tissue engineering [13,14,15,16].

In particular, mesoporous silicas (MSs) gained increasing attention as inert carriers in different drug delivery applications thanks to their fundamental characteristics such as high specific surface area, adsorption capacity for therapeutic agents and excellent biocompatibility. Moreover, the presence of a high concentration of silanol groups (Si-OH) on their surface can favor a large number of surface reactions [17,18,19,20,21,22]. One of the first attempts to use MSs as carriers for drugs has been reported by Vallet-Regí and co-workers, who demonstrated the ability of MCM-41-type MSs to incorporate ibuprofen into their pores and subsequently release it in simulated body fluids [23]. The high loading capability shown by MSs can be attributed to their high pore volume as well as long-range ordered and highly accessible mesoporous structures [18,24,25]. The pore features in terms of size and shape are essential factors in determining the drug loading capacity and release kinetics [26]. However, MSs synthesized using an amphiphilic triblock copolymer made of poly(ethylene oxide) and poly(propylene oxide) (PEO–PPO–PEO) as a surfactant (e.g., Pluronic P123 or Pluronic F127) are normally characterized by nanopores ranging between 2–8 nm [27], which are unsuitable when the adsorption of large and bulky biomolecules such as proteins, enzymes, and growth factors (GFs) is desired. To solve this limitation, MSs with larger pores (LPMSs) up to 18–20 nm have been produced by adding cosolvents [28], block-co-polymers with long hydrophobic chains [29], or more frequently swelling agents [30,31,32]. Pore expanders such as 1,3,5-trimethylbenzene (TMB) [33,34,35,36,37,38,39], ethylbenzene [40], decane [41,42,43], hexane [44,45], heptane [36,46], nonane [46], 1,3,5-triisopropylbenzene (TIPB)/cyclohexane [47], and octane [48] are able to dissolve into the hydrophobic cores of the micelles, leading to their enlargement then obtaining larger pores upon template removal. In parallel, current strategies often consider the use of high temperature hydrothermal treatments to widen the mesopores while enhancing the long-range order of the meso-structure when using highly hydrophilic surfactants (e.g., Pluronic F127) [49,50]. Furthermore, some studies showed that the adsorption and structural properties of large-pore mesoporous materials can be tailored by manipulating the hydrothermal treatment parameters, such as temperature and process time [51,52,53,54].

The increased size of pores of LPMSs materials can allow their use in the field of bone tissue engineering as carriers for the incorporation and release of bone GFs, with the final aim of stimulating bone regeneration in compromised healing situations. As a matter of fact, bone tissue is characterized by a continuous turnover, known as bone remodeling, where bone resorption by osteoclasts (OCs) is sequentially coupled with new bone deposition by osteoblasts (OBs) [55,56]. During the bone resorption phase, active OCs arrange their cytoskeleton to form an adhesion belt-like structure which defines a bone area underneath, known as the “sealing zone” [57]. The pH underneath this sealed region drops to 5.0–5.5 due to H^+^ species secreted by OCs, generating a local acidic microenvironment that, in combination with the OC secreted enzymes (proteases), is responsible for the bone matrix degradation and the subsequent release of the encased GFs [57]. The latter regulate a variety of cellular processes, including the stimulation of OBs recruitment and activity [58].

In this context, LPMSs coated with pH-responsive materials can potentially mimic these physiological mechanisms, enabling the smart release of biomolecules previously incorporated into mesopores with a suitable size. Among the synthetic polymers, poly(ethylene glycol) (PEG) is currently one of the most popular used in the biomedical field due to its proven biocompatibility and widely reported “stealth” properties [59,60]. PEG specifically belongs to the class of polyethers and is extensively used as a solvent, plasticizer, surfactant and ointment to obtain a wide variety of products such pharmaceuticals [61], cosmetics [62], lubricants [63], and inks [64]. It is also frequently used as bioconjugation agent to prolong blood circulation time and improve drug efficacy due to its shielding effect on the surface charge of DDSs [65]. Furthermore, PEG was extensively exploited as a surface-coating polymer for DDS particles to prevent non-specifically proteins interaction, improve sterically stability, and prevent premature release of the incorporated therapeutic agents [66,67,68]. Surprisingly, the literature about its use as pH-sensitive coating for inorganic particles is still limited, however, few studies clearly proved the faster release of the encased biomolecules at acidic pH compared to neutral or basic conditions, when PEG was exploited as coating layer [69,70].

Based on the previous observations, in the present work, LPMSs able to incorporate large molecular weight proteins have been developed by using TMB as an expanding agent. A hydrothermal treatment was optimized, investigating the potential influence of different temperatures and times on the final mesostructure and pore size. Afterwards, the adsorption capability and the release of a large-protein model, horseradish peroxidase (HRP), from the optimized LPMSs were explored.

With the purpose of achieving a smart release of the incorporated HRP by mimicking the local pH decrease provoked by the resorption activity of OCs, the developed LPMSs were coated with PEG as a pH-responsive polymer able to degrade at acidic pH and trigger the biomolecule release. To this aim, a silane functionalized PEG (mPEG-silane) able to covalently bind its triethoxyl silane moieties to the hydroxyl groups on silica particle surface has been employed [71].

Finally, with the objective of designing a 3D printed scaffold for bone regeneration applications, the developed PEG-coated carriers were combined with type I collagen to develop a hybrid formulation, as a proof of concept. The authors previously explored this procedure by incorporating into collagen-based solutions both strontium-containing mesoporous bioactive glasses (MBGs) and nanometric hydroxyapatite particles (nHA), to develop constructs able to reproduce the natural composition of bone tissue [72,73,74,75]. In this work, the authors aim to extend the existing knowledge on collagenous composites by embedding the optimized PEG-coated LPMS loaded with HRP in a collagen matrix, allowing the fabrication of scaffolds able to mimic the physiological release of these biomolecules during bone resorption.

## 2. Materials and Methods

### 2.1. Preparation of Large-Pore Mesoporous Silica Particles

LPMSs were synthesized combining the use of a swelling agent with a hydrothermal method and the influence of the different process temperatures and duration on pore size and morphology has been investigated.

#### 2.1.1. Synthesis of Mesoporous Silica-Based Particles with Large Pores (LPMSs)

LPMSs particles were synthesized by hydrothermal-assisted procedure under acidic conditions by using a commercially available triblock copolymer (Pluronic F127, Sigma-Aldrich, Milan, Italy) as micellar template and TMB (Mesitylene, 98%, Sigma-Aldrich, Milan, Italy) as micelle expander adapting the procedure reported by Ma et al. [76].

Briefly, 0.5 g of Pluronic F127 and 0.6 g of TMB were mixed with 50 mL of 1M hydrochloric acid (HCl) and the solution was stirred for 1 h at room temperature (RT) to allow micelles self-assembly. Then, 2.08 g of silica source (tetraethyl orthosilicate—TEOS, >99% (GC), Sigma-Aldrich, Milan, Italy) was added and the mixture was kept under agitation at RT for 1 day. Subsequently, the obtained suspension was transferred in a tightly closed polypropylene bottle and heated at the chosen temperature (100 °C–140 °C–220 °C) for two different durations: 2 h or 24 h. The produced wet precipitate was collected by centrifugation (Hermle Labortechnik Z326, Hermle LaborTechnik GmbH, Wehingen, Germany) at 10,000 rpm for 5 min. Then, it was washed four times with 70% ethanol solution in order to remove all acidic residuals, checking the pH at each wash step, and subsequently dried at 70° C overnight. Finally, a calcination step (Carbolite 1300 CWF 15/5, Carbolite Ltd., Hope Valley, UK) was performed at 550 °C under air for 6 h (heating rate of 1.5 °C/min) to remove the surfactant template and stabilize the silica framework. The resulting samples were named as LPMS-X_t, where X denotes the hydrothermal treatment temperature expressed in °C and *t* the process duration expressed in hours.

#### 2.1.2. Characterization of LPMSs

The synthesized materials were characterized in terms of specific surface area, pore volume, pore size distribution, and morphology.

Nitrogen adsorption-desorption isotherms were measured at −196 °C by using Micromeritics ASAP 2020 Plus Physisorption instrument (Norcross, GA, USA). Before the analysis, about 150 mg of each sample was outgassed under vacuum at 150 °C for 4 h. The Brunauer–Emmett–Teller (BET) equation was used to calculate the specific surface area (S_BET_) value. The pore size distribution was determined through the Density Functional Theory (DFT) method using the Non-Local DFT (NLDFT) kernel of equilibrium isotherms on the desorption branch.

The LPMSs morphology was observed by Field-Emission Scanning Electron Microscopy (FE-SEM—ZEISS MERLIN instrument, Oberkochen, Germany) operated at 3 kV. Before FE-SEM imaging, a 7 nm thick Pt layer was sputtered on particles previously dispersed on a conductive carbon tape in order to increase sample conductivity.

### 2.2. Study of Protein Adsorption and Release

The ability of the LPMS_140_24 to adsorb and release large biomolecules was evaluated using HRP as a model protein due to molecular size and charge properties similar to those of GFs [77].

#### 2.2.1. Procedure for HRP Adsorption into LPMS_140_24 Mesopores (LPMS-HRP)

The adsorption of horseradish peroxidase (HRP) into LPMS-140_24 mesopores was carried out by adapting the method reported by Chouyyok et al. [78]. First, a protein solution at a concentration of 1 mg/mL was prepared in Phosphate-Buffered Saline (PBS, pH 7.4). Thereafter, 100 mg of LPMS_140_24 was mixed with 2 mL of protein solution and gently stirred at 4 °C for 24 h. Next, the obtained HRP-loaded particles (LPMS-HRP) were collected by centrifugation at 12,000 rpm for 5 min and the supernatant was kept and stored at −20 °C for subsequent adsorption efficiency test. LPMS-HRP powders were dried under laminar airflow for 48 h and then stored under vacuum at 4 °C for further studies.

#### 2.2.2. Evaluation of the Effective HRP Adsorption

The effective HRP adsorption into LPMS_140_24 mesopores was assessed through different analyses.

About 150 mg of dry powder, previously outgassed under vacuum at 25 °C for 4 h, was investigated by using nitrogen physisorption instrument to determine the pore volume reduction and evaluate changes in the isotherm shape. An outgassing temperature of 25 °C has been used to avoid the denaturation of the loaded protein.

Fourier transform infrared spectroscopy in attenuated total reflectance mode (ATR-FTIR) was performed to assess the effective presence of HRP. ATR-FTIR spectra were collected between 4000 and 600 cm^−1^ at 4 cm^−1^ resolution using 32 scans using an Equinox 55 spectrometer (Bruker, Ettlingen, Germany) equipped with an MCT cryodetector and an ATR accessory. The spectra were reported after background subtraction, baseline correction, and smoothing (11 points) using OPUS software (Bruker, Ettlingen, Germany).

The amount of adsorbed HRP, called adsorption efficiency (*AE*%), was calculated using the formula below:

$$AE\%=\frac{\left(p_{total}-p_{free}\right)}{p_{total}}\;\%$$

where *p_free_* is the concentration of HRP in the supernatant after centrifugation and *p_total_* is the initial concentration of HRP in the solution. The concentration of protein in solution was determined by using a MicroBCA Assay Kit (ThermoFisher, Milan, Italy) following the manufacturer’s instructions and the absorbance was measured at 562 nm using a spectrophotometer (MultiscanGo, Thermo Scientific, Waltham, MA, USA). Experiments were performed in triplicate. Wide-angle (2θ within 15–80°) X-ray diffraction measurements (XRD—X’Pert PRO, PANalytical, Malvern, UK) were performed using CuKα radiation at 40 kV and 40 mA to evaluate the amorphous status of the adsorbed HRP.

#### 2.2.3. In Vitro Protein Release Test

The release kinetics of adsorbed HRP was determined by soaking 10 mg of LPMS_HRP in 2 mL of PBS, pH 7.4 [21,79]. The resulting suspension was placed in an orbital shaker (Excella E24, Eppendorf, Hamburg, Germany) at 37 °C with an agitation rate of 150 rpm. At specified time points (1 h, 3 h, 5 h, 7 h, 24 h), the entire volume of supernatant was withdrawn after a centrifugation step (6000 rpm, 3 min), stored at −20 °C and fully replaced with fresh buffer.

The amount of released HRP was assessed by a MicroBCA Assay Kit as described above. All measurements were carried out in triplicate.

### 2.3. pH-Responsive Surface Coating of LPMS-HRP

With the purpose of releasing HRP upon pH drop, mPEG-silane (average Mn 5000, Sigma-Aldrich, Milan, Italy) was covalently grafted on the outer surface of LPMS-HRP in order to form a pH-sensitive layer.

#### 2.3.1. Preparation and Characterization of PEG-Coated LPMS-HRP via PEGylation (LPMS-HRP_PEG)

**Figure 1:**
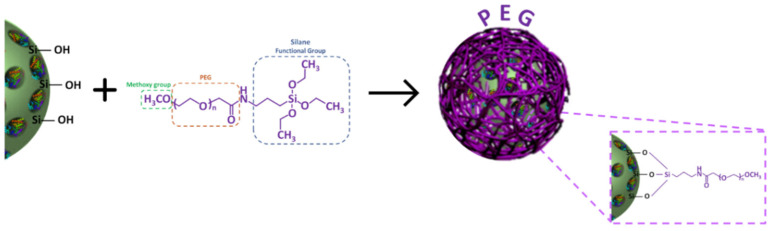
Schematic representation of the coating of mPEG-silane on LPMS_HRP obtained through PEGylation method.

LPMS-HRP particles were covered with mPEG-silane through a PEGylation method. The reaction occurred in 95% ethanol and the ratio LPMS-HRP/PEG was set to maximize the reaction between hydroxyl groups on LPMS surface and triethoxysilane moieties of mPEG-silane (Figure 1) [80]. In detail, a PEG stock solution at a concentration of 40 mg/mL was prepared by dissolving the polymer in 95% ethanol for 30 min. Then, 150 mg of LPMS-HRP were dispersed in 5 mL of 95% ethanol followed by the addition of 5 mL PEG stock solution reaching a final concentration of polymer of 20 mg/mL. After stirring for 1 h at 4 °C, the particles were collected through centrifugation (9000 rpm, 5 min) and washed three times with distilled water (dH_2_O). The supernatants recovered after each centrifugation step were stored at −20 °C for further investigations.

Lastly, PEG-coated LPMS-HRP (LPMS-HRP_PEG) were let dry under laminar airflow for 48 h and then stored under vacuum at 4°C for subsequent assessments.

FE-SEM, nitrogen physisorption and ATR-FTIR analyses were carried out with the aim of evaluating the effective PEG coating on LPMS-HRP particles.

FE-SEM images were acquired on powders sputtered with a 7 nm platinum layer. Adsorption-desorption isotherms were obtained by outgassing the samples for 4 h at 25 °C to avoid the damage of protein and PEG coating.

ATR-FTIR spectra were collected between 4000 and 600 cm^−1^ at 4 cm^−1^ resolution using 32 scans as previously described. Furthermore, thermogravimetric analysis (TGA) (TGA/SDTA851^e^, Mettler Toledo, Columbus, OH, USA) was performed in order to evidence the presence of PEG coating on LPMS-HRP by monitoring the weight loss. TGA curves of LPMS-HRP_PEG, LPMS-HRP and LPMS as such were collected over a temperature range of 25–1000 °C with a heating rate of 10 °C/min under air in a flow of 50 mL/min.

Lastly, a MicroBCA assay was performed on the reaction and washing solutions after PEGylation to determine the amount of HRP potentially released during the grafting process. Experiments were analyzed in triplicate following the instructions of the manufacturer.

#### 2.3.2. In Vitro HRP Release Kinetics at pH 5.5 and pH 7.4 from LPMS-HRP_PEG Carriers

The in vitro release of HRP from PEG-coated LPMS was investigated under neutral and acidic conditions and compared with those obtained from bare LPMS-HRP particles, by adapting the method reported by Totovao and Dhivya et al. [69,80].

LPMS-HRP_PEG was suspended in 2 mL PBS both at pH 7.4 and pH 5.5 (PBS with 2M HCl) at a final concentration of 5 mg/mL. All suspensions were placed in an orbital shaker at 37 °C, 150 rpm up to 24 h. At defined time points (1 h, 3 h, 5 h, 7 h, 24 h), the whole volume of supernatants was removed after centrifugation (6000 rpm, 3 min) and substituted with the same quantity of fresh buffer.

The amount of released HRP was quantified by means of a MicroBCA Assay kit measuring the absorbance at 562 nm. Two separated standard curves using PBS pH 7.4 and 5.5 as medium were prepared to measure HRP concentration in neutral and acidic solutions, respectively. The experiments were carried out in triplicates.

### 2.4. 3D Printing of the Collagen-Based Composite Systems

With the final goal of designing a 3D printed scaffold for bone applications, as proof of concept, a composite formulation was developed by incorporating LPMS-HRP_PEG into a type I collagen matrix and related rheology and printability have been assessed.

#### 2.4.1. Preparation of the Composite Suspension Based on Collagen and LPMS-HRP_PEG

A collagen solution was obtained dissolving type I collagen powders (type I collagen from bovine Achilles tendon, Blafar Ltd., Dublin, Ireland) in 0.5 M acetic acid stirring overnight at 4 °C. After complete dissolution, the pH was neutralized by adding 1 M sodium hydroxide (NaOH) [73]. LPMS-HRP_PEG particles were dispersed in dH_2_O and subsequently added to the neutralized collagen solution to obtain a homogenous system with a final collagen concentration of 1.5 wt%. The weight amounts of collagen powders and LPMS-HRP_PEG particles were calculated in order to be aligned with the volume percentages of the organic and inorganic phases in the natural bone tissue (53% *v*/*v* and 47% *v*/*v*, respectively) [81].

To promote the enzymatic crosslinking of the collagenous system upon simil-physiological conditions (37 °C, pH 7.4), transglutaminase powders from guinea pig (TG, Sigma Aldrich, Milan, Italy) were added to the resulting formulation. In detail, TG powders were firstly dissolved in a solution of 2 mM DL-dithiothreitol (DTT), 5 mM calcium chloride (CaCl_2_) and 10 mM Tris (pH 7.0) in dH_2_O, and subsequently added to the collagen-based composite suspension kept at 10 °C to reach a final concentration of 50 μg/mL [82,83,84]. The suspension containing TG was stirred for about 15 min and immediately used for analysis and 3D printing. The entire process was carried out at 10 °C to avoid premature gelation of the collagenous suspension and the final composite system obtained will be further reported as Coll/LPMS-HRP_PEG_TG.

#### 2.4.2. Rheological Characterization

The printability and the overall visco-elastic properties of the collagenic formulation were assessed by means of a DHR-2 controlled stress rotational rheometer (TA Instruments, Waters, Milan, Italy) equipped with a controlled temperature system (Peltier plate) using a 20 mm parallel plate geometry.

A flow ramp test was performed to study the variation of the suspension viscosity for increasing shear rates applied (10^−2^–10^3^ s^−1^) keeping a constant temperature of 10 °C. The stability of the visco-elastic properties, expressed as storage (G′) and loss (G″) moduli, at 10 °C and the sol-gel transition of the system at 37 °C were observed through time sweep tests carried out at 1% strain and 1 Hz for 1 h.

#### 2.4.3. 3D Printing of 3D Mesh-Like Composite Scaffolds

3D printed mesh-like scaffolds (10 × 10 × 3 mm^3^) were printed using a temperature-controlled pneumatic printhead installed on a 3D BIOX Bioprinter (CELLINK, Gothenburg, Sweden).

Based on the FRESH method (Freeform Reverse Embedding of Suspended Hydrogels) [75,85], the Coll/LPMS-HRP_PEG_TG suspension was kept at 10 °C and printed in a gelatin-slurry kept at 20 °C to support the deposition of the extruded filaments and improve the printing fidelity and the overall final resolution of the constructs. The suspension was previously gently centrifuged before printing and the scaffolds were obtained selecting a mesh-like structure with a 15% infill and using 27 G needles (200 μm internal diameter) after the proper optimization of the printing parameters. After printing, the scaffolds were incubated overnight at 37 °C in order to promote the physical and enzymatic crosslinking of the system while enabling the removal of the gelatin-based bath. The scaffolds were washed two times in dH_2_O to remove any residue of gelatin and stored at 4 °C until further investigations.

To perform the morphological analysis, the scaffolds were lyophilized for 24 h, coated with a 7 nm thick Pt layer, and analyzed by FE-SEM at an accelerating voltage of 5 kV.

## 3. Results and Discussion

### 3.1. LPMS Particles Characterization

All the developed LPMS particles were characterized in terms of specific surface area (S_BET_), pore size distribution, pore volume, and morphology.

**Figure 2:**
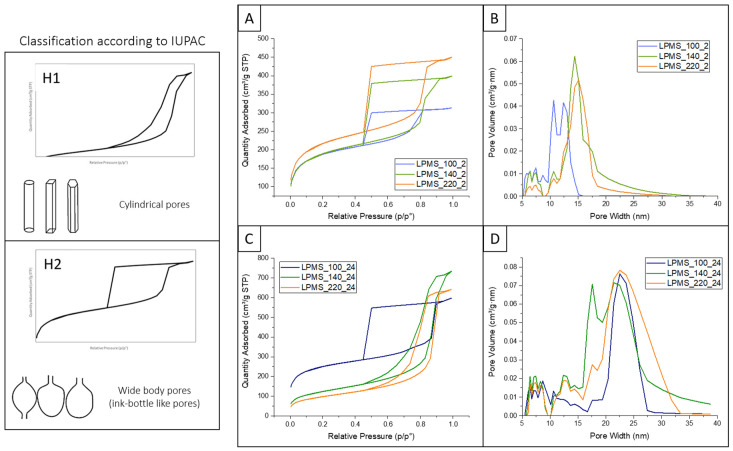
N_2_ adsorption-desorption isotherms and pore size distributions of synthesized LPMSs using different temperatures and durations of the hydrothermal treatment: LPMSs obtained with 2 h (**A**,**B**) and 24 h (**C**,**D**) of process, respectively. The classification of hysteresis loop according to pore types is reported to the left (according to reference [86]).

The isotherms of LPMS materials were evaluated by N_2_ adsorption/desorption measurements as reported in Figure 2, while Table 1 summarizes the textural properties of the synthesized LPMS samples.

As shown in Figure 2, the hydrothermal temperature and treatment time proved to have a significant effect on the final S_BET_, pore volume, and pore mean diameter. All materials revealed a high S_BET_ and a type IV isotherm, typical of the mesoporous materials [86], with different hysteresis loop shapes. The presence of the hysteresis loop is ascribed to the capillary condensation phenomenon which takes place in mesopores while the shape is closely related to the pore structure and underlying adsorption mechanism. The different temperatures and durations of the process resulted in a change of hysteresis loop shape between H1 and H2 (according to IUPAC classification) [86].

In details, LPMSs synthesized by a hydrothermal treatment of 2 h at any tested temperatures (100 °C, 140 °C, 220 °C) exhibited the characteristic Type H2 hysteresis loop and uniform mesopore size distribution (Figure 2A,B). Type H2 are representative of ink-bottle shaped pores, characterized by an entrance smaller than the size of the pore body. A small neck of the pore is considered unsuitable for the adsorption of large biomolecules that would mostly remain exposed at the pore entrance, causing an extensive pore blocking. Despite LPMS_100_24 sample showing a similar isotherm curve, they presented a larger average pore width of about 23 nm, indicating that a prolonged hydrothermal treatment is beneficial to obtain pores with bigger diameters. By observing the isotherms resulting from the analysis on LPMS_140_24 and LPMS_220_24, a change from Type H2 to Type H1 hysteresis loop has been pointed out. LPMS_140_24 and LPMS_220_24 samples presented a narrow hysteresis loop, indicating a cylindrical or columnar form of the pores (Figure 2C). Moreover, the shift of desorption branches towards high relative pressure indicates an enlargement of the pore entrance dimension. As a matter of fact, LPMS_140_24 e LPMS_220_24 exhibited higher pore diameters and larger pore volume: 1.09 and 0.96 cm^3^/g, respectively. Nevertheless, a decrease in the S_BET_ from 834 to 348 m^2^/g was observed when both temperature and duration of the hydrothermal process were increased. This can be ascribed to the partial destruction and collapse of the pore structures due to the instability of Pluronic F127 micelles at high temperatures [87].

These results suggested that an extended process at high temperature allowed to obtain larger pores with a cylinder-like morphology, greater volume but lower S_BET_. Accordingly, other studies reported that the increase of the hydrothermal temperature is associated with a noteworthy narrowing of the hysteresis loop and an enlargement of pore size [88,89,90]. These effects are mostly due to the temperature influence on Pluronic F127 structure during the hydrothermal process. Pluronic F127 is an amphiphilic triblock copolymer composed of hydrophobic PPO and hydrophilic PEO segments. As the temperature increases, the hydrophilicity of the PEO block decreases due to its dehydration, resulting in a larger hydrophobic micellar core and the subsequent increase of the pore size. Furthermore, the geometry of micelles changes from spherical to cylindrical or rod-like according to the temperature increase, due to the greater interactions between PEO blocks of close micelles [91]. However, the use of temperatures higher than 140 °C can compromise the overall degree of order, leading to the destruction and collapse of pore structure and detected by a significant reduction in the S_BET_ [92].

**Figure 3:**
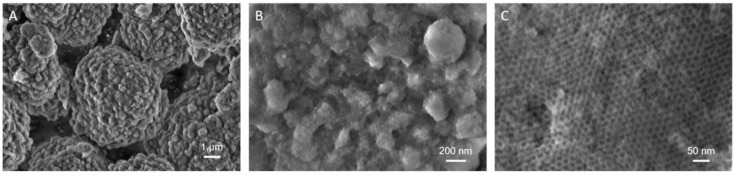
FE-SEM images of LPMS_140_24 at different magnifications: single particles (**A**), surface cage-like mesoporous network (**B**,**C**).

The morphological assessment on LPMSs produced with a hydrothermal treatment of 2 h showed an irregular morphology and particles with irregular dimensions, as illustrated in Supplementary Materials Figure S1A,C,E. LPMSs obtained from 24 h of hydrothermal process exhibited a near-spherical morphology with particle dimensions between 7–10 μm (Figure S1G,I and Figure 3A). LPMS_140_24 particles showed mostly spherical morphologies with relatively uniform sizes of 5–7 μm (Figure 3A). Moreover, at higher magnification images indicated that this material exhibited a cage-like pores network throughout the surface of the particles (Figure 3B,C), indicating a highly ordered and uniform three-dimensional pore framework. The cage diameter directly calculated from the FE-SEM images was about 20 nm, in good accordance with the N_2_ desorption/NLDFT results.

Based on the presented results, LPMS_140_24 powders were selected for further experiments since they showed the higher pore volume and the best compromise in terms of pore diameter, pore volume, S_BET_ and morphology.

### 3.2. HRP Absorption Efficiency Assessment and In Vitro Release Test

The ability of LPMS_140_24 particles to adsorb and release large molecular weight molecules was assessed using horseradish peroxidase (HRP) as model proteins.

As observed in Figure S2, the isotherm of LPMS-HRP particles showed a less pronounced slope desorption curve compared to LPMS_140_24 samples (Figure 2C), likely related to a restriction of mesoporous channels due to the successful incorporation of the biomolecules on the inner surface of LPMS-140-24 particles. This observation was underlined by the pore size distribution in which the peak at 23 nm, shown by the bare samples, was no longer visible (Figure S2). Moreover, the total pore volume decreases from 1.09 to 0.8 cm^3^/g, confirming that a great fraction of mesopores was occupied by the incorporated HRP molecules.

**Figure 4:**
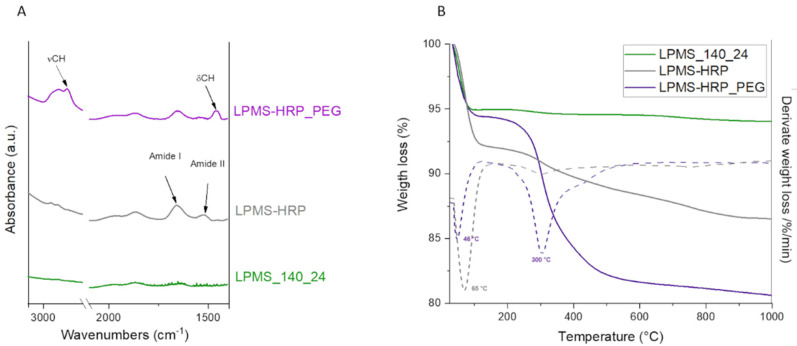
ATR-FTIR spectrum (**A**) and thermogravimetric analysis (TGA) curves (**B**) with relative derivative TGA curve (dotted line) of LPMS_140_24 (green), LPMS-HRP (grey), and LPMS-HRP_PEG (purple) particles.

The ATR-FTIR spectra of LPMS-HRP specimen reported in Figure 4A showed new peaks due to the presence of HRP at 1670 cm^−1^ and 1540 cm^−1^ corresponding to amide I and amide II, respectively, confirming the adsorption of HRP into LPMS_140_24 mesopores.

The adsorption efficiency (AE%) was calculated through MicroBCA Assay by determining the concentration of HRP in the supernatant after 24 h of adsorption at 4 °C and resulted to be equal to 81.1 ± 0.1%. The high surface area exhibited from LPMS_140_24 and the open structure of their pores allowed high loading efficiency. The incorporated protein was proved to be in an amorphous status since no diffraction peaks were observed by XRD (Figure S3) and this is known to be an essential feature for promoting an effective release of the protein.

**Figure 5:**
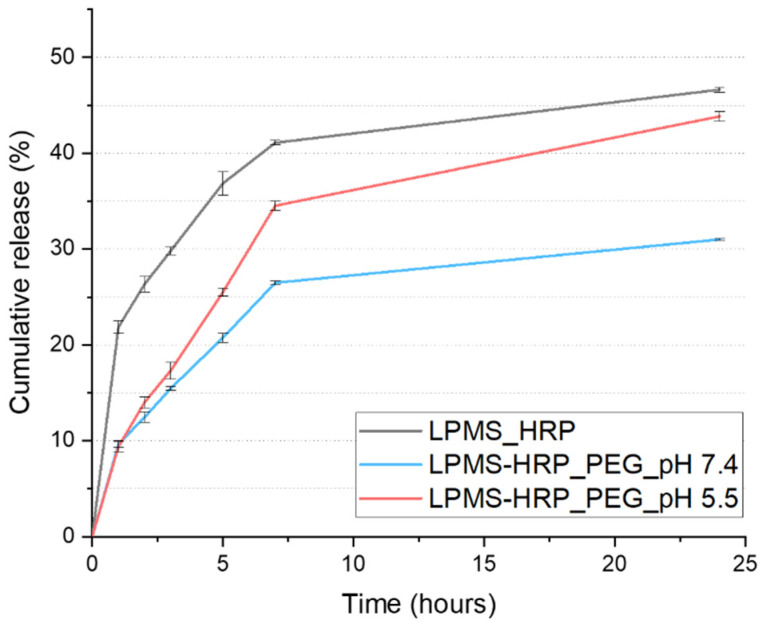
Horseradish peroxidase (HRP) release profiles from LPMS-HRP particles under physiological conditions (grey), PEG-coated samples at pH 7.4 (light blue) and pH 5.5 (light red).

In vitro release test was performed at 37 °C, and pH 7.4 up to 24 h and the release kinetics of HRP from LPMS_140_24 is reported in Figure 5. The results showed that about 21.8% of the absorbed protein was released in the first hour, which is probably due to the presence of a certain amount of protein located at the mesopore entrances. After 7 h, HRP was progressively released registering 41% of the overall amount, reaching 46.6% after 24 h. The slower release rate observed between 7 and 24 h might be imputable to the release of protein molecules located deeply into the mesopores, characterized by a higher number of interactions with the silica surface.

The obtained results thus proved the successful incorporation of proteins as HRP into large mesoporous silica-based particles by adsorption method and their subsequent release.

### 3.3. PEG Coating Assessment and In Vitro Evaluation of the Triggering Release Kinetics in Acidic Conditions

With the aim to trigger the release of the biomolecules exploiting a pH drop, in an attempt to mimic the growth factors release mechanism caused by the resorption activity of OCs during bone remodeling [55,56,57], LPMS-HRP particles were coated with a pH-sensitive PEG layer.

**Figure 6:**
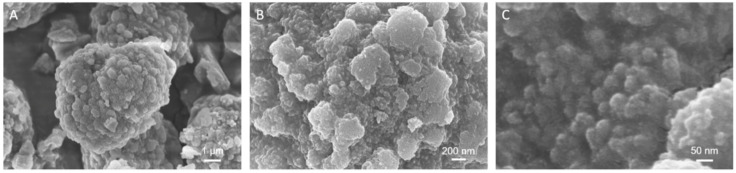
FE-SEM images of LPMS-HRP_PEG material at low (**A**) and high (**B**,**C**) magnifications.

FE-SEM images at high magnifications demonstrated that the cage-like porous network on LPMS_140_24 surface almost disappeared after the PEGylation process and a smooth area, imputable to the polymeric coating, was observed (Figure 6B,C). However, the spherical morphology of the particles and their dimensions were maintained, as shown in Figure 6A. The successful PEG-coating has also been proved by the decrease in S_BET_ and pores volume of LPMS-HRP_PEG samples compared with particles as such: from 438 to 335 m^2^/g and from 0.8 to 0.6 cm^3^/g, respectively. Furthermore, in comparison to LPMS_140_24 material, the polymeric coating caused a change in the hysteresis loop shape from Type H1 to Type H2 (Figure S4). These results suggested that the hydroxyl groups on the surface of LPMSs reacted with the silane groups of mPEG-silane and the mesopores were partially capped by the polymeric coating.

The presence of the PEG coating was further confirmed by means of ATR-FTIR and TGA analyses. After surface modification, peaks for C-H stretching at about 2900 cm^−1^ and C-H bending at 1460 cm^−1^ were observed on ATR-FTIR spectra of LPMS-HRP_PEG particles (Figure 4A), indicating the presence of -CH_2_- groups related to the repetitive units of mPEG grafted on silica particles [93].

Furthermore, the mass percentage of grafted PEG relative to the total mass of the materials has been determined by means of TGA analysis (Figure 4B). A significant weight loss has been observed from 250 °C until 600 °C and can be ascribed to the decomposition of PEG. In particular, examining the derivative TGA curve (dotted line), PEG started to degrade at about 300 °C, in accordance with data reported in the literature [94]. An additional weight loss in the range of 25–100 °C due to the release of the surface, adsorbed water and water bound to the protein was noted for both loaded and coated samples. The additional weight loss in the temperature range between 100 and 300 °C, observed exclusively for LPMS-HRP material, can be assigned to the denaturation of the loaded protein.

MicroBCA Assay on PEGylation solution supernatants revealed the 14.8 ± 0.07% of the loaded HRP was lost during the coating process, showing that 1 h can be a suitable period to realize the covalent bonding between silica particles and PEG alongside minimizing the waste of the loaded protein.

The pH-responsive release of HRP due to PEG coating was investigated at pH 5.5 and 7.4, simulating the local acidic environment under the resorption zone formed by OCs during bone remodeling [55] and the normal physiological environment, respectively. Typically, a pH drop under the OCs sealing zone is lower than the pH value of not resorbed tissue [56,57], meaning that the release of HRP from LPMS-HRP_PEG carriers would be triggered by the acidic condition present in the resorption lacuna. Figure 5 depicts the cumulative percentage of released HRP as a function of time.

The release kinetics of HRP have been thus registered in acidic conditions up to 24 h to avoid HRP denaturation due to the direct and prolonged contact of the biomolecule with the acidic solution [69,80]. Slower release kinetics were obtained from LPMS-HRP_PEG samples compared with LPMS-HRP, indicating that the polymeric coating enabled to significantly reduce the initial burst release observed for bare particles. Indeed, the concentration of the protein after 1 h of immersion was found to be 9.6% and 9.4% when coated carriers were soaked at pH 7.4 and pH 5.5, respectively, in comparison with 21.8% calculated for LPMS-HRP. This result confirmed the proper coating of silica-based material with the polymeric layer that acts as a barrier to protein diffusion. Afterwards, the pH-responsive behavior of PEG has been proved since a higher concentration of HRP was calculated for carriers tested in acidic medium, as illustrated in Figure 5. About 34.5% and 26.5% of HRP was released after 7 h from LPMS-HRP_PEG at pH 5.5 and pH 7.4, respectively, reaching values of 43.8% and 31% of release at 24 h. The faster release of HRP in acidic conditions can be ascribed to the degradation mechanisms of PEG catalyzed by the protonation of the carbonyl oxygen, leading to accelerated hydrolysis of acetal linkages.

These findings are consistent with the few studies reported in the literature about the use of PEG as pH-responsive polymer for inorganic particles coating. For instance, Dhivya and co-workers observed that the curcumin release rate from their developed platform based on ZnO nanoparticles coated with PEG and poly(methyl methacrylate) (PMMA) after 30 h of soaking at 37 °C was faster at pH 5.4 than in physiological conditions [69]. The faster degradation of PEG under acidic conditions has been confirmed also by Stillman et al., who noted that polymeric nanoparticles made of poly(ethylene glycol)diacrylate (PEGDA) upon a month of immersion at pH 5.0, 37 °C lost more than half of the initial sample mass, at variance with neutral or basic conditions (pH 10.0), which allowed slower degradation kinetics [70].

### 3.4. 3D Printing of the Collagen-Based Composite System

To prove the potential use of the developed carriers in the design of more complex 3D structures, LPMS-HRP_PEG particles have been combined with type I collagen to create a formulation suitable for the 3D extrusion printing of scaffolds.

Based on previous studies conducted by the authors [73,75], a homogeneous suspension was produced by dispersing LPMS-HRP_PEG particles into a highly concentrated collagen solution at neutral pH in order to avoid the premature dissolution of the PEG coating, as well as promoting the self-assembly of collagen in simil-physiological conditions (37 °C; pH 7.4). Moreover, TG was added in the final formulation in order to promote the enzymatic crosslinking of collagen and further improve the stability of the system.

**Figure 7:**
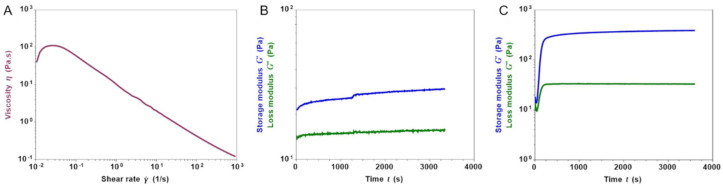
Shear thinning (**A**) and stability of the storage (G′) and loss (G″) modulus values of the Coll/LPMS-HRP_PEG_TG suspension over time (**B**) at 10 °C. Sol-gel transition of the composite system at 37 °C (**C**).

The printability of the composite formulation (Coll/LPMS-HRP_PEG_TG) was firstly assessed by means of rheological analyses, testing the visco-elastic properties of the developed material at different conditions. As represented in Figure 7A, the viscosity of the suspension kept at 10 °C significantly decreased from about 110 Pa.s down to 0.12 Pa.s when increasing shear rates were applied, proving the shear thinning behavior of the system, a key feature for the material inks used for extrusion printing technologies [95,96]. In addition, the time sweep test performed at 10 °C (Figure 7B) further confirmed the processability and stability of the material, showing no evident changes in the storage (G′) and loss (G″) modulus values over time, even if TG was included in the initial formulation of the system.

Despite the presence of the inorganic particles, the self-assembly of the collagen molecules and the enzymatic crosslinking was proved (Figure 7C). Keeping the system at 37 °C, the rapid increase of G′ over G″ showed the formation of a stable gel up to 1 h and reaching values of about 386 Pa and 33 Pa, respectively.

**Figure 8:**
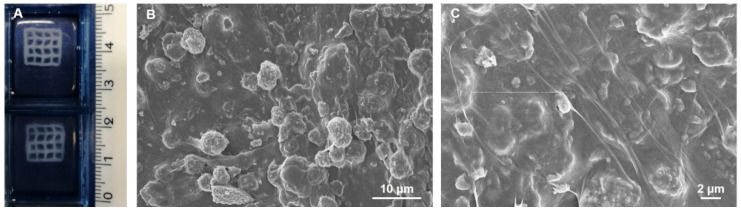
3D mesh-like scaffolds of Coll/LPMS-HRP_PEG_TG (**A**) and FE-SEM images representing their micro-structures at different magnifications (**B**,**C**).

According to the rheological results, the use of the developed formulation as material ink for extrusion printing technologies was additionally proved by obtaining 3D mesh-like scaffolds presenting a surface of 10 × 10 mm^2^ and a thickness up to 3 mm (Figure 8A). The 3D printed scaffolds were produced exploiting a strategy previously optimized by the authors and based on the FRESH method [75,85]. After the proper optimization of the printing parameters, the 3D printed composite constructs showed a high-resolution mesh-like structure with a great printing fidelity. In particular, a pressure of 30 kPa, a printhead speed of 8 mm/s and a z-layer of 170 μm were selected to obtain the best resolution and printing fidelity. After printing, the constructs were incubated overnight at 37 °C in order to promote the sol-gel transition of the system due to the natural self-assembly of collagen and the enzymatic crosslinking by TG [75,82,83,84]. Furthermore, the gel to sol transition of gelatin at 37 °C enabled the easy removal of the printing supporting bath.

FE-SEM images performed on lyophilized scaffolds (Figure 8B,C) proved the homogeneous distribution and embedding of the LPMS-HRP_PEG particles into the collagenous matrix at the microscale, together with the successful reconstitution of a highly fibrillar structure due to the collagen crosslinking.

The biocompatibility of mesoporous silicas used as DDS, PEG as coating, and bovine type I collagen as 3D support for cells is widely demonstrated both in vitro and in vivo [97,98,99].

## 4. Conclusions

In this study, the successful development of mesoporous silica microparticles with large pores up to 23 nm (LPMSs) synthesized via an optimized hydrothermal treatment has been reported. For a final application in the field of bone tissue engineering, the optimized LPMS carriers were coated with a pH-sensitive PEG-based polymer, with the aim of triggering the release of the incorporated protein by exploiting a pH decrease. In particular, the designed smart system aims at mimicking the mechanism underlaying the physiological release of GFs encased in the bone matrix upon the local pH drop caused by the osteoclast resorption activity during bone remodeling.

The tuning of synthesis parameters, particularly the temperature and duration of the hydrothermal process, allowed to obtain mesoporous particles with the desired characteristics. This enabled the generation of open, large pores with dimensions greater than 20 nm. LPMS synthesized at 140 °C for 24 h was found to be the best candidate for the adsorption of large biomolecules since it presented the best compromise in term of specific surface area, mesostructure framework, pore size, and entrance dimension. A protein model, HRP, has been successfully adsorbed into LPMS mesopores and the release kinetics at different pH (pH 7.4 and pH 5.5) from the silica microparticles as such and after PEGylation have been investigated. PEG-coated carriers tested at acidic pH enabled a faster release compared to those observed under physiological conditions.

Finally, PEGylated LPMS have been incorporated into a type I collagen suspension to produce a biomaterial ink for the design of high-resolution 3D printed scaffolds to be used in the field of bone tissue engineering.

These findings lay the basis for future studies focused on the development of biomimetic stimuli-responsive systems able to modulate the release of biomolecules that could find several biomedical applications (such as bone tissue engineering, treatment of osteolytic tumor lesions) where the acidic conditions can trigger the release of an incorporated biomolecule.

Further investigations are ongoing using bone GFs (e.g., insulin-like growth factor 1—IGF1 and transforming growth factor Beta1—TGF-β1) and Western blot analysis will be performed to exclude chemical and structural damages during the entire manufacturing process.

## Figures and Tables

**Table 1:** Specific surface area (S_BET_), pore volume and average pore size of synthesized LPMS materials.

Sample	S_BET_ (m^2^/g)	Pore Volume (cm^3^/g)	Pores Mean Diameter (nm)
LPMS_100_2	834	0.49	10–12
LPMS_140_2	634	0.60	14
LPMS_220_2	536	0.70	16
LPMS_100_24	626	0.93	23
LPMS_140_24	428	1.09	17–23
LPMS_200_24	348	0.96	23

## Data Availability

Data is contained within the article or supplementary materials.

## Supplementary Materials

The following are available online at https://www.mdpi.com/1422-0067/22/4/1718/s1, Figure S1: FE-SEM images of the different synthesized LPMS particles and their surface pores network: LPMS_100_2 (**A**,**B**), LPMS_140_2 (**C**,**D**), LPMS_220_2 (**E**,**F**), LPMS_100_24 (**G**,**H**), LPMS_220_24 (**I**,**L**). Figure S2: N_2_ adsorption-desorption isotherm and pore size distribution of LPMS-HRP material. Figure S3: XRD pattern of LPMS_140_24 material before (green) and after HRP adsorption (grey). Figure S4: N_2_ adsorption-desorption isotherm and pore size distribution of LPMS-HRP_PEG material.

## References

[1] Witharana C., Wanigasekara J. (2017). Drug Delivery Systems: A New Frontier in Nano-Technology. Int. J. Med. Res. Health Sci..

[2] Tiwari G., Tiwari R., Bannerjee S., Bhati L., Pandey S., Pandey P., Sriwastawa B. (2012). Drug Delivery Systems: An Updated Review. Int. J. Pharm. Investig..

[3] Heller J. (1980). Controlled Release of Biologically Active Compounds from Bioerodible Polymers. Biomater. Silver Jubil. Compend..

[4] Narayan R., Nayak U.Y., Raichur A.M., Garg S. (2018). Mesoporous Silica Nanoparticles: A Comprehensive Review on Synthesis and Recent Advances. Pharmaceutics.

[5] Shi Z., Zhou Y., Fan T., Lin Y., Zhang H., Mei L. (2020). Inorganic Nano-Carriers Based Smart Drug Delivery Systems for Tumor Therapy. Smart Mater. Med..

[6] Quignard S., Coradin T., Powell J.J., Jugdaohsingh R. (2017). Silica Nanoparticles as Sources of Silicic Acid Favoring Wound Healing in Vitro. Colloids Surf. B Biointerfaces.

[7] Hamam F., Nasr A. (2020). Curcumin-Loaded Mesoporous Silica Particles as Wound-Healing Agent: An In Vivo Study. Saudi J. Med. Med. Sci..

[8] Pan Z., Zhang K.R., Gao H.L., Zhou Y., Yan B.B., Yang C., Zhang Z.Y., Dong L., Chen S.M., Xu R. (2020). Activating Proper Inflammation for Wound-Healing Acceleration via Mesoporous Silica Nanoparticle Tissue Adhesive. Nano Res..

[9] Iturrioz-Rodríguez N., Correa-Duarte M.A., Fanarraga M.L. (2019). Controlled Drug Delivery Systems for Cancer Based on Mesoporous Silica Nanoparticles. Int. J. Nanomed..

[10] Arap W., Pasqualini R., Montalti M., Petrizza L., Prodi L., Rampazzo E., Zaccheroni N., Marchiò S. (2013). Luminescent Silica Nanoparticles for Cancer Diagnosis. Curr. Med. Chem..

[11] Vivero-Escoto J.L., Huxford-Phillips R.C., Lin W. (2012). Silica-Based Nanoprobes for Biomedical Imaging and Theranostic Applications. Chem. Soc. Rev..

[12] Shirshahi V., Soltani M. (2015). Solid Silica Nanoparticles: Applications in Molecular Imaging. Contrast Media Mol. Imaging.

[13] Izquierdo-Barba I., Colilla M., Vallet-Regí M. (2008). Nanostructured Mesoporous Silicas for Bone Tissue Regeneration. J. Nanomater..

[14] Chen L., Zhou X., He C. (2019). Mesoporous Silica Nanoparticles for Tissue-Engineering Applications. Wiley Interdiscip. Rev. Nanomed. Nanobiotechnol..

[15] Shadjou N., Hasanzadeh M. (2015). Silica-Based Mesoporous Nanobiomaterials as Promoter of Bone Regeneration Process. J. Biomed. Mater. Res. Part. A.

[16] Gisbert-Garzarán M., Manzano M., Vallet-Regí M. (2020). Mesoporous Silica Nanoparticles for the Treatment of Complex Bone Diseases: Bone Cancer, Bone Infection and Osteoporosis. Pharmaceutics.

[17] Bitar A., Ahmad N.M., Fessi H., Elaissari A. (2012). Silica-Based Nanoparticles for Biomedical Applications. Drug Discov. Today.

[18] Zhou Y., Quan G., Wu Q., Zhang X., Niu B., Wu B., Huang Y., Pan X., Wu C. (2018). Mesoporous Silica Nanoparticles for Drug and Gene Delivery. Acta Pharm. Sin. B.

[19] Knežević N., Durand J.O. (2015). Large Pore Mesoporous Silica Nanomaterials for Application in Delivery of Biomolecules. Nanoscale.

[20] Jafari S., Derakhshankhah H., Alaei L., Fattahi A., Varnamkhasti B.S., Saboury A.A. (2019). Mesoporous Silica Nanoparticles for Therapeutic/Diagnostic Applications. Biomed. Pharmacother..

[21] Xia W., Chang J. (2006). Well-Ordered Mesoporous Bioactive Glasses (MBG): A Promising Bioactive Drug Delivery System. J. Control. Release.

[22] Labouta H.I., Schneider M. (2013). Interaction of Inorganic Nanoparticles with the Skin Barrier: Current Status and Critical Review. Nanomed. Nanotechnol. Biol. Med..

[23] Vallet-Regi M., Rámila A., Del Real R.P., Pérez-Pariente J. (2001). A New Property of MCM-41: Drug Delivery System. Chem. Mater..

[24] Juère E., Kleitz F. (2018). On the Nanopore Confinement of Therapeutic Drugs into Mesoporous Silica Materials and Its Implications. Microporous Mesoporous Mater..

[25] Farjadian F., Roointan A., Mohammadi-Samani S., Hosseini M. (2019). Mesoporous Silica Nanoparticles: Synthesis, Pharmaceutical Applications, Biodistribution, and Biosafety Assessment. Chem. Eng. J..

[26] Rosenholm J.M., Lindén M. (2008). Towards Establishing Structure-Activity Relationships for Mesoporous Silica in Drug Delivery Applications. J. Control. Release.

[27] Izquierdo-Barba I., Vallet-Regi M. (2015). Mesoporous Bioactive Glasses: Relevance of Their Porous Structure Compared to That of Classical Bioglasses. Biomed. Glas..

[28] Niu D., Ma Z., Li Y., Shi J. (2010). Synthesis of Core-Shell Structured Dual-Mesoporous Silica Spheres with Tunable Pore Size and Controllable Shell Thickness. J. Am. Chem. Soc..

[29] Chen Y., Xu P., Chen H., Li Y., Bu W., Shu Z., Li Y., Zhang J., Zhang L., Pan L. (2013). Colloidal HPMO Nanoparticles: Silica-Etching Chemistry Tailoring, Topological Transformation, and Nano-Biomedical Applications. Adv. Mater..

[30] Kim M.H., Na H.K., Kim Y.K., Ryoo S.R., Cho H.S., Lee K.E., Jeon H., Ryoo R., Min D.H. (2011). Facile Synthesis of Monodispersed Mesoporous Silica Nanoparticles with Ultralarge Pores and Their Application in Gene Delivery. ACS Nano.

[31] Lee C.H., Lin T.S., Mou C.Y. (2009). Mesoporous Materials for Encapsulating Enzymes. Nano Today.

[32] Carlsson N., Gustafsson H., Thörn C., Olsson L., Holmberg K., Åkerman B. (2014). Enzymes Immobilized in Mesoporous Silica: A Physical-Chemical Perspective. Adv. Colloid Interface Sci..

[33] Nguyen T.P.B., Lee J.W., Shim W.G., Moon H. (2008). Synthesis of Functionalized SBA-15 with Ordered Large Pore Size and Its Adsorption Properties of BSA. Microporous Mesoporous Mater..

[34] Kruk M. (2012). Access to Ultralarge-Pore Ordered Mesoporous Materials through Selection of Surfactant/Swelling-Agent Micellar Templates. Acc. Chem. Res..

[35] Schmidt-winkel P., Lukens W.W., Zhao D., Yang P., Chmelka B.F., Stucky G.D., Barbara S. (2000). Mesocellular Siliceous Foams with Uniformly Sized Cells and Windows Molecular Sieves with Uniform Large Pores Are Desirable for Chemical Reactions and for Use in Separations Involving Large Molecules. 1 Periodic Cubic and Hexagonal Mesoporous Silica Phas. J. Am. Chem. Soc..

[36] Ma J., Liu Q., Chen D., Wen S., Wang T. (2015). Synthesis and Characterisation of Pore-Expanded Mesoporous Silica Materials. Micro Nano Lett..

[37] Blin J.L., Su B.L. (2002). Tailoring Pore Size of Ordered Mesoporous Silicas Using One or Two Organic Auxiliaries as Expanders. Langmuir.

[38] Xin C., Zhao N., Zhan H., Xiao F., Wei W., Sun Y. (2014). Phase Transition of Silica in the TMB-P123-H2O-TEOS Quadru-Component System: A Feasible Route to Different Mesostructured Materials. J. Colloid Interface Sci..

[39] Wei J., Wang H., Deng Y., Sun Z., Shi L., Tu B., Luqman M., Zhao D. (2011). Solvent Evaporation Induced Aggregating Assembly Approach to Three-Dimensional Ordered Mesoporous Silica with Ultralarge Accessible Mesopores. J. Am. Chem. Soc..

[40] Huang L., Kruk M. (2012). Synthesis of Ultra-Large-Pore FDU-12 Silica Using Ethylbenzene as Micelle Expander. J. Colloid Interface Sci..

[41] Zhang H., Sun J., Ma D., Bao X., Klein-Hoffmann A., Weinberg G., Su D., Schlögl R. (2004). Unusual Mesoporous SBA-15 with Parallel Channels Running along the Short Axis. J. Am. Chem. Soc..

[42] Egger S.M., Hurley K.R., Datt A., Swindlehurst G., Haynes C.L. (2015). Ultraporous Mesostructured Silica Nanoparticles. Chem. Mater..

[43] Blin J.L., Otjacques C., Herrier G., Su B.L. (2000). Pore Size Engineering of Mesoporous Silicas Using Decane as Expander. Langmuir.

[44] Shan W., Wang W., Ru H. (2015). Siliceous Mesocellular Foams Modified via a Partitioned Cooperative Self-Assembly Process Using Hexane as Pore Swelling Agent. J. Non. Cryst. Solids.

[45] Boahene P.E., Soni K.K., Dalai A.K., Adjaye J. (2011). Application of Different Pore Diameter SBA-15 Supports for Heavy Gas Oil Hydrotreatment Using FeW Catalyst. Appl. Catal. A Gen..

[46] Sun J., Zhang H., Ma D., Chen Y., Bao X., Klein-Hoffmann A., Pfänder N., Su D.S. (2005). Alkanes-Assisted Low Temperature Formation of Highly Ordered SBA-15 with Large Cylindrical Mesopores. Chem. Commun..

[47] Cao L., Kruk M. (2010). Synthesis of Large-Pore SBA-15 Silica from Tetramethyl Orthosilicate Using Triisopropylbenzene as Micelle Expander. Colloids Surf. A Physicochem. Eng. Asp..

[48] Sun J., Zhang H., Tian R., Ma D., Bao X., Su D.S., Zou H. (2006). Ultrafast Enzyme Immobilization over Large-Pore Nanoscale Mesoporous Silica Particles. Chem. Commun..

[49] Feng P., Bu X., Pine D.J. (2000). Control of Pore Sizes in Mesoporous Silica Templated by Liquid Crystals in Block Copolymer-Cosurfactant-Water Systems. Langmuir.

[50] Man T. (2014). Design of Well-Defined Mesoporous Silicas via Surfactant Templating Method Enhanced by the Use of Swelling Agents.

[51] Lawrence G., Baskar A.V., El-Newehy M.H., Cha W.S., Al-Deyab S.S., Vinu A. (2015). Quick High-Temperature Hydrothermal Synthesis of Mesoporous Materials with 3D Cubic Structure for the Adsorption of Lysozyme. Sci. Technol. Adv. Mater..

[52] Lei C., Shin Y., Liu J., Ackerman E.J. (2002). Entrapping Enzyme in a Functionalized Nanoporous Support. J. Am. Chem. Soc..

[53] Hwang Y.K., Lee K.C., Kwon Y.U. (2001). Nanoparticle Routes to Mesoporous Titania Thin Films. Chem. Commun..

[54] Aksay I.A., Trau M., Manne S., Honma I., Yao N., Zhou L., Fenter P., Eisenberger P.M., Gruner S.M. (1996). Biomimetic Pathways for Assembling Inorganic Thin Films. Science.

[55] Borciani G., Montalbano G., Baldini N., Cerqueni G., Vitale-Brovarone C., Ciapetti G. (2020). Co–Culture Systems of Osteoblasts and Osteoclasts: Simulating in Vitro Bone Remodeling in Regenerative Approaches. Acta Biomater..

[56] Licini C., Vitale-Brovarone C., Mattioli-Belmonte M. (2019). Collagen and Non-Collagenous Proteins Molecular Crosstalk in the Pathophysiology of Osteoporosis. Cytokine Growth Factor Rev..

[57] Takito J., Inoue S., Nakamura M. (2018). The Sealing Zone in Osteoclasts: A Self-Organized Structure on the Bone. Int. J. Mol. Sci..

[58] Florencio-Silva R., da Silva Sasso G.R., Sasso-Cerri E., Simões M.J., Cerri P.S. (2015). Biology of Bone Tissue: Structure, Function, and Factors That Influence Bone Cells. Biomed. Res. Int..

[59] Howard M.D., Jay M., Dziubla T.D., Lu X. (2008). PEGylation of Nanocarrier Drug Delivery Systems: State of the Art. J. Biomed. Nanotechnol..

[60] Joralemon M.J., McRae S., Emrick T. (2010). PEGylated Polymers for Medicine: From Conjugation to Self-Assembled Systems. Chem. Commun..

[61] D’souza A.A., Shegokar R. (2016). Polyethylene Glycol (PEG): A Versatile Polymer for Pharmaceutical Applications. Expert Opin. Drug Deliv..

[62] Jang H.-J., Shin C.Y., Kim K.-B. (2015). Safety Evaluation of Polyethylene Glycol (PEG) Compounds for Cosmetic Use. Toxicol. Res..

[63] Kobayashi M., Koide T., Hyon S.H. (2014). Tribological Characteristics of Polyethylene Glycol (PEG) as a Lubricant for Wear Resistance of Ultra-High-Molecular-Weight Polyethylene (UHMWPE) in Artificial Knee Join. J. Mech. Behav. Biomed. Mater..

[64] Sun Q., Luo Y., Xiang P., Yang X., Shen M. (2017). Analysis of PEG Oligomers in Black Gel Inks: Discrimination and Ink Dating. Forensic Sci. Int..

[65] Thi T.T.H., Pilkington E.H., Nguyen D.H., Lee J.S., Park K.D., Truong N.P. (2020). The Importance of Poly(Ethylene Glycol) Alternatives for Overcoming PEG Immunogenicity in Drug Delivery and Bioconjugation. Polymers.

[66] Dombb A., Miillerd R.H., Langerf R., Gref R., Domb A., Quellec P., Blunk T., Müller R.H., Verbavatz J.M., Langer R. (1995). The Controlled Intravenous Delivery of Drugs Using PEG-Coated Sterically Stabilized Nanospheres. Adv. Drug Deliv. Rev..

[67] Bunker A. (2012). Poly (Ethylene Glycol) in Drug Delivery, Why Does It Work, and Can We Do Better? All Atom Molecular Dynamics Simulation Provides Some Answers. Phys. Procedia.

[68] Fam S.Y., Chee C.F., Yong C.Y., Ho K.L., Mariatulqabtiah A.R., Tan W.S. (2020). Stealth Coating of Nanoparticles in Drug-Delivery Systems. Nanomaterials.

[69] Dhivya R., Ranjani J., Bowen P.K., Rajendhran J., Mayandi J., Annaraj J. (2017). Biocompatible Curcumin Loaded PMMA-PEG/ZnO Nanocomposite Induce Apoptosis and Cytotoxicity in Human Gastric Cancer Cells. Mater. Sci. Eng. C.

[70] Stillman Z., Jarai B.M., Raman N., Patel P., Fromen C.A. (2020). Degradation Profiles of Poly (Ethylene Glycol)Diacrylate (PEGDA)-Based Hydrogel Nanoparticles. Polym. Chem..

[71] Gidi Y., Bayram S., Ablenas C.J., Blum A.S., Cosa G. (2018). Efficient One-Step PEG-Silane Passivation of Glass Surfaces for Single-Molecule Fluorescence Studies. ACS Appl. Mater. Interfaces.

[72] Montalbano G., Fiorilli S., Caneschi A., Vitale-Brovarone C. (2018). Type I Collagen and Strontium-Containing Mesoporous Glass Particles as Hybrid Material for 3D Printing of Bone-like Materials. Materials.

[73] Montalbano G., Borciani G., Pontremoli C., Ciapetti G., Mattioli-Belmonte M., Fiorilli S., Vitale-Brovarone C. (2019). Development and Biocompatibility of Collagen-Based Composites Enriched with Nanoparticles of Strontium Containing Mesoporous Glass. Materials.

[74] Montalbano G., Molino G., Fiorilli S., Vitale-Brovarone C. (2020). Synthesis and Incorporation of Rod-like Nano-Hydroxyapatite into Type I Collagen Matrix: A Hybrid Formulation for 3D Printing of Bone Scaffolds. J. Eur. Ceram. Soc..

[75] Montalbano G., Borciani G., Cerqueni G., Licini C., Banche-Niclot F., Janner D., Sola S., Fiorilli S., Mattioli-Belmonte M., Ciapetti G. (2020). Collagen Hybrid Formulations for the 3D Printing of Nanostructured Bone Scaffolds: An Optimized Genipin-Crosslinking Strategy. Nanomaterials.

[76] Ma G., Yan X., Li Y., Xiao L., Huang Z., Lu Y., Fan J. (2010). Ordered Nanoporous Silica with Periodic 30-60 Nm Pores as an Effective Support for Gold Nanoparticle Catalysts with Enhanced Lifetime. J. Am. Chem. Soc..

[77] Gajhede M., Schuller D.J., Henriksen A., Smith A.T., Poulos T.L. (1997). Crystal Structure of Horseradish Peroxidase C at 2.15 Å Resolution. Nat. Struct. Biol..

[78] Chouyyok W., Panpranot J., Thanachayanant C., Prichanont S. (2009). Effects of PH and Pore Characters of Mesoporous Silicas on Horseradish Peroxidase Immobilization. J. Mol. Catal. B Enzym..

[79] Tu J., Boyle A.L., Friedrich H., Bomans P.H.H., Bussmann J., Sommerdijk N.A.J.M., Jiskoot W., Kros A. (2016). Mesoporous Silica Nanoparticles with Large Pores for the Encapsulation and Release of Proteins. ACS Appl. Mater. Interfaces.

[80] Totovao R. (2017). Stimuli-Responsive Breakable Hybrid Organic/Inorganic Silica Nanoparticles for Biomedical Applications. Ph.D. Thesis.

[81] Schwarcz H.P., Abueidda D., Jasiuk I. (2017). The Ultrastructure of Bone and Its Relevance to Mechanical Properties. Front. Phys..

[82] Orban J.M., Wilson L.B., Kofroth J.A., El-Kurdi M.S., Maul T.M., Vorp D.A. (2004). Crosslinking of Collagen Gels by Transglutaminase. J. Biomed. Mater. Res. Part. A.

[83] Fortunati D., Chau D.Y.S., Wang Z., Collighan R.J., Griffin M. (2014). Cross-Linking of Collagen I by Tissue Transglutaminase Provides a Promising Biomaterial for Promoting Bone Healing. Amino Acids.

[84] Chau D.Y.S., Collighan R.J., Verderio E.A.M., Addy V.L., Griffin M. (2005). The Cellular Response to Transglutaminase-Cross-Linked Collagen. Biomaterials.

[85] Hinton T.J., Jallerat Q., Palchesko R.N., Park J.H., Grodzicki M.S., Shue H.J., Ramadan M.H., Hudson A.R., Feinberg A.W. (2015). Three-Dimensional Printing of Complex Biological Structures by Freeform Reversible Embedding of Suspended Hydrogels. Sci. Adv..

[86] Sing K.S.W., Everett D.H., Haul R.A.W., Moscou L., Pierotti R.A., Rouquerol J., Siemieniewska T. (1985). Reporting Physisorption Data for Gas/Solid Systems with Special Reference to the Determination of Surface Area and Porosity. Pure Appl. Chem..

[87] Dou Q., Karim A.A., Loh X.J. (2016). Modification of Thermal and Mechanical Properties of PEG-PPG-PEG Copolymer (F127) with MA-POSS. Polymers.

[88] Fan J., Lie J., Wang L., Yu C., Tu B., Zhao D. (2003). Rapid and High-Capacity Immobilization of Enzymes Based on Mesoporous Silicas with Controlled Morphologies. Chem. Commun..

[89] Washmon-Kriel L., Jimenez V.L., Balkus K.J. (2000). Cytochrome c Immobilization into Mesoporous Molecular Sieves. J. Mol. Catal. B Enzym..

[90] Mazinani B., Beitollahi A., Masrom A.K., Ibrahim S., Jamil F. (2012). The Effect of Aging Temperature on the Pores of Mesoporous SBA-15 Silica. AIP Conf. Proc..

[91] Song H.M., Zink J.I. (2019). Ag(i)-Mediated Self-Assembly of Anisotropic Rods and Plates in the Surfactant Mixture of CTAB and Pluronics. RSC Adv..

[92] Mortensen K., Pedersen J.S. (1993). Structural Study on the Micelle Formation of Poly (Ethylene Oxide)–Poly (Propylene Oxide)–Poly(Ethylene Oxide) Triblock Copolymer in Aqueous Solution. Macromolecules.

[93] Catauro M., Bollino F., Nocera P., Piccolella S., Pacifico S. (2016). Entrapping Quercetin in Silica/Polyethylene Glycol Hybrid Materials: Chemical Characterization and Biocompatibility. Mater. Sci. Eng. C.

[94] Seker A., Arslan B., Chen S. (2019). Recovery of Polyphenols from Grape Pomace Using Polyethylene Glycol (PEG)-Grafted Silica Particles and PEG-Assisted Cosolvent Elution. Molecules.

[95] Paxton N., Smolan W., Böck T., Melchels F., Groll J., Jungst T. (2017). Proposal to Assess Printability of Bioinks for Extrusion-Based Bioprinting and Evaluation of Rheological Properties Governing Bioprintability. Biofabrication.

[96] Schwab A., Levato R., D’Este M., Piluso S., Eglin D., Malda J. (2020). Printability and Shape Fidelity of Bioinks in 3D Bioprinting. Chem. Rev..

[97] Tang F., Li L., Chen D. (2012). Mesoporous Silica Nanoparticles: Synthesis, Biocompatibility and Drug Delivery. Adv. Mater..

[98] Alcantar N.A., Aydil E.S., Israelachvili J.N. (2000). Polyethylene Glycol-Coated Biocompatible Surfaces. J. Biomed. Mater. Res..

[99] Pasqua L., De Napoli I.E., De Santo M., Greco M., Catizzone E., Lombardo D., Montera G., Comandè A., Nigro A., Morelli C. (2019). Mesoporous Silica-Based Hybrid Materials for Bone-Specific Drug Delivery. Nanoscale Adv..

