# Supplementary material for: PEG-Coated Large Mesoporous Silicas as Smart Platform for Protein Delivery and Their Use in a Collagen-Based Formulation for 3D Printing
Source: Int J Mol Sci. 2021 Feb 9;22(4):1718. doi: 10.3390/ijms22041718 (PMC7914545; doi:10.3390/ijms22041718)
Supplement: Supplementary file 1 [file ijms-22-01718-s001.zip › SI and Figure LPMS_revised_300dpi/SI_Manuscript LPMS-PEG_revised.docx]

Supporting Information

PEG-Coated Large Mesoporous Silicas as Smart Platform for Protein Delivery and Their Use in a Collagen-Based Formulation for 3D Printing

Federica Banche-Niclot ^1, 2^, Giorgia Montalbano ^1^, Sonia Fiorilli ^1,3^ and Chiara Vitale-Brovarone ^1,3^ *

^1^ Department of Applied Science and Technology, Politecnico di Torino, 10029 Torino, Italy; federica.banche@polito.it (F. B.-N.); giorgia.montalbano@polito.it (G. M.); sonia.fiorilli@polito.it (S. F.); chiara.vitale@polito.it (C.V.-B.)

^2^ Department of Surgical Science, Università degli Studi di Torino, 10029 Torino, Italy

^3^ National Interuniversity Consortium of Materials Science and Technology (RU Politecnico di Torino)

***** Correspondence: chiara.vitale@polito.it (C.V.-B.)

Received: date; Accepted: date; Published: date


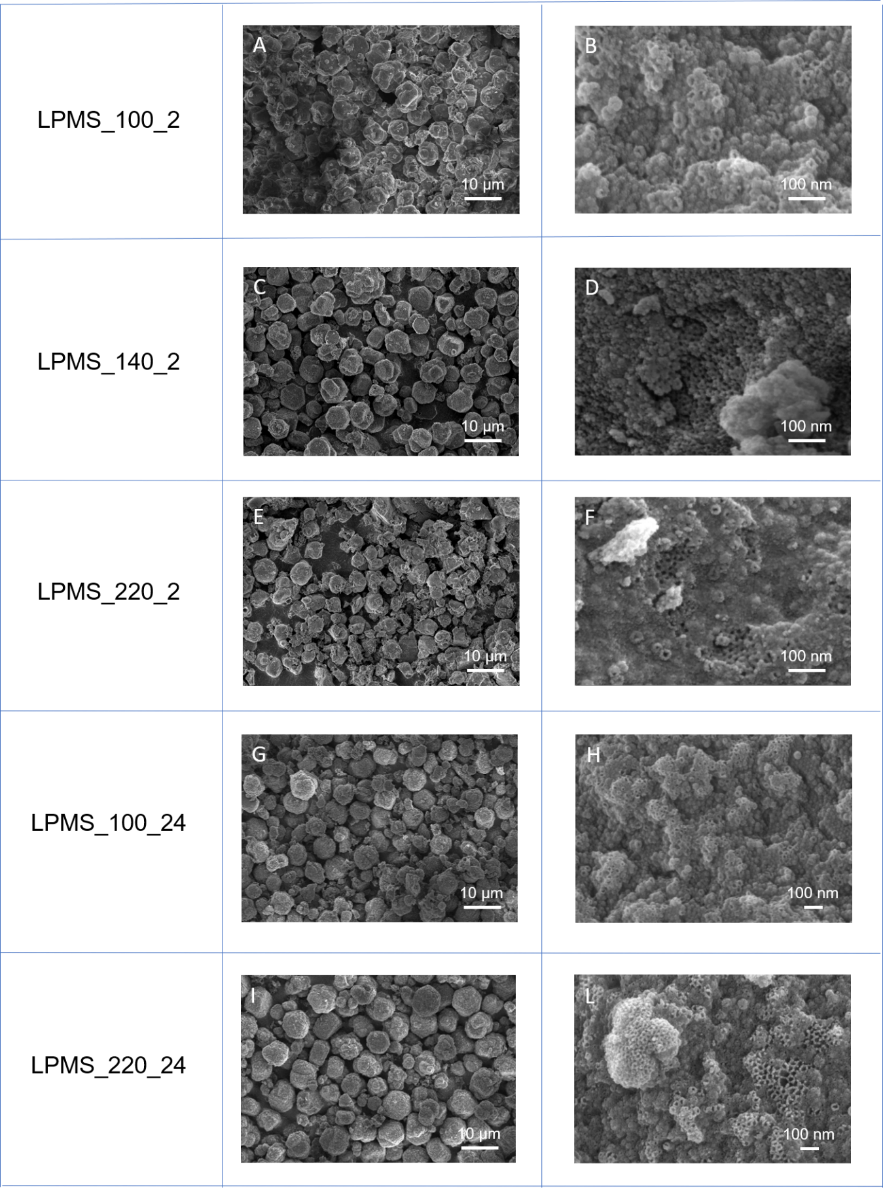


**Figure S1.** *FE-SEM images of the different synthesised LPMS particles and their surface pores network: LPMS_100_2 (A, B), LPMS_140_2 (C, D), LPMS_220_2 (E, F), LPMS_100_24 (G, H), LPMS_220_24 (I, L).*


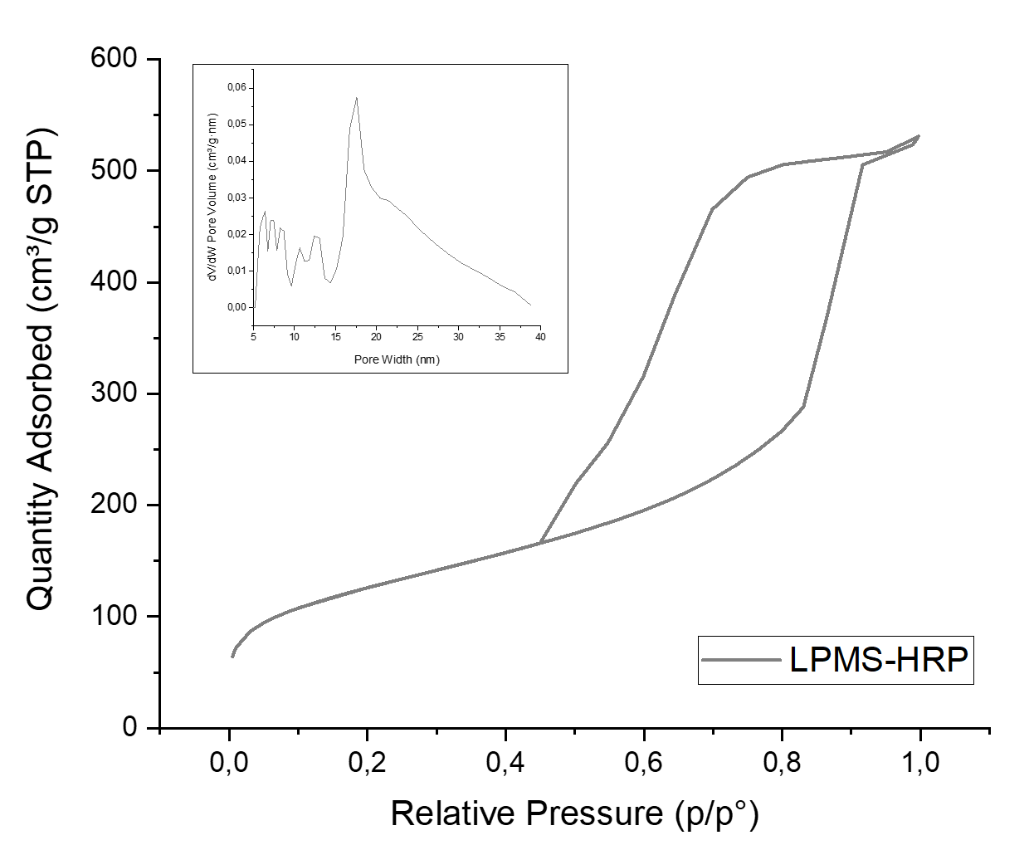


**Figure S2*.*** *N_2_ adsorption-desorption isotherm and pore size distribution of LPMS-HRP material.*


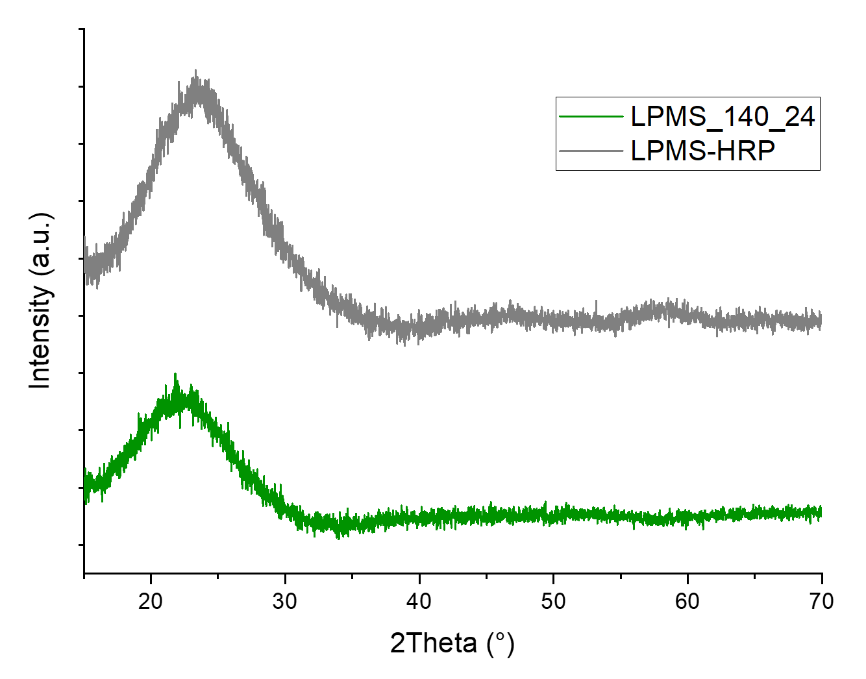


**Figure S3*.*** *XRD pattern of LPMS_140_24 material before (green) and after HRP adsorption (grey).*


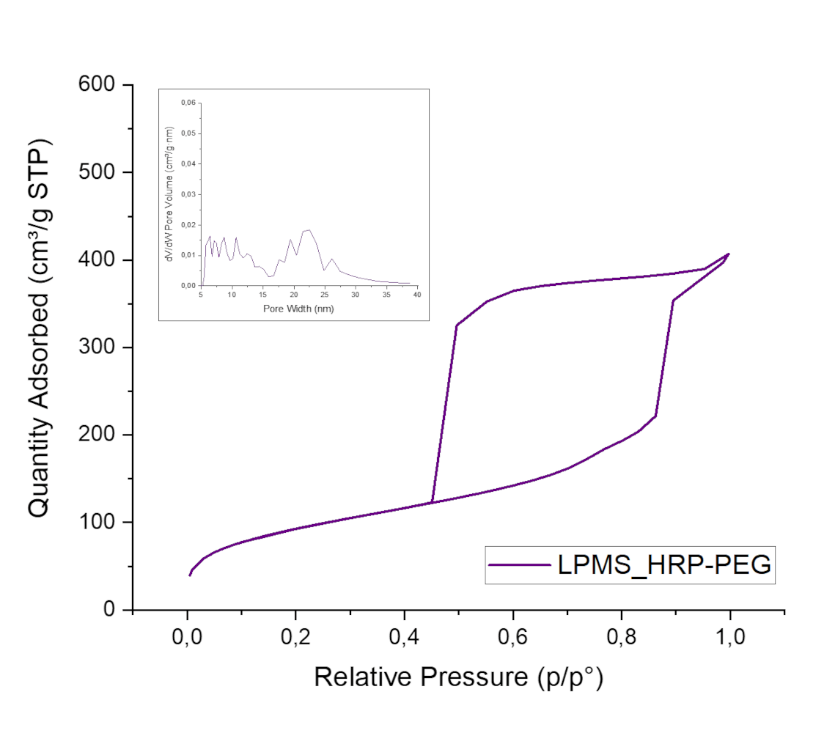


**Figure S4*.*** *N_2_ adsorption-desorption isotherm and pore size distribution of LPMS-HRP_PEG material.*

| 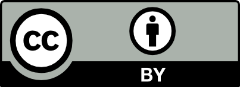 | © 2020 by the authors. Submitted for possible open access publication under the terms and conditions of the Creative Commons Attribution (CC BY) license (http://creativecommons.org/licenses/by/4.0/). |
| --- | --- |
