# Supplementary figures and images for: PEG-Coated Large Mesoporous Silicas as Smart Platform for Protein Delivery and Their Use in a Collagen-Based Formulation for 3D Printing
Source: Int J Mol Sci. 2021 Feb 9;22(4):1718. doi: 10.3390/ijms22041718 (PMC7914545; doi:10.3390/ijms22041718)

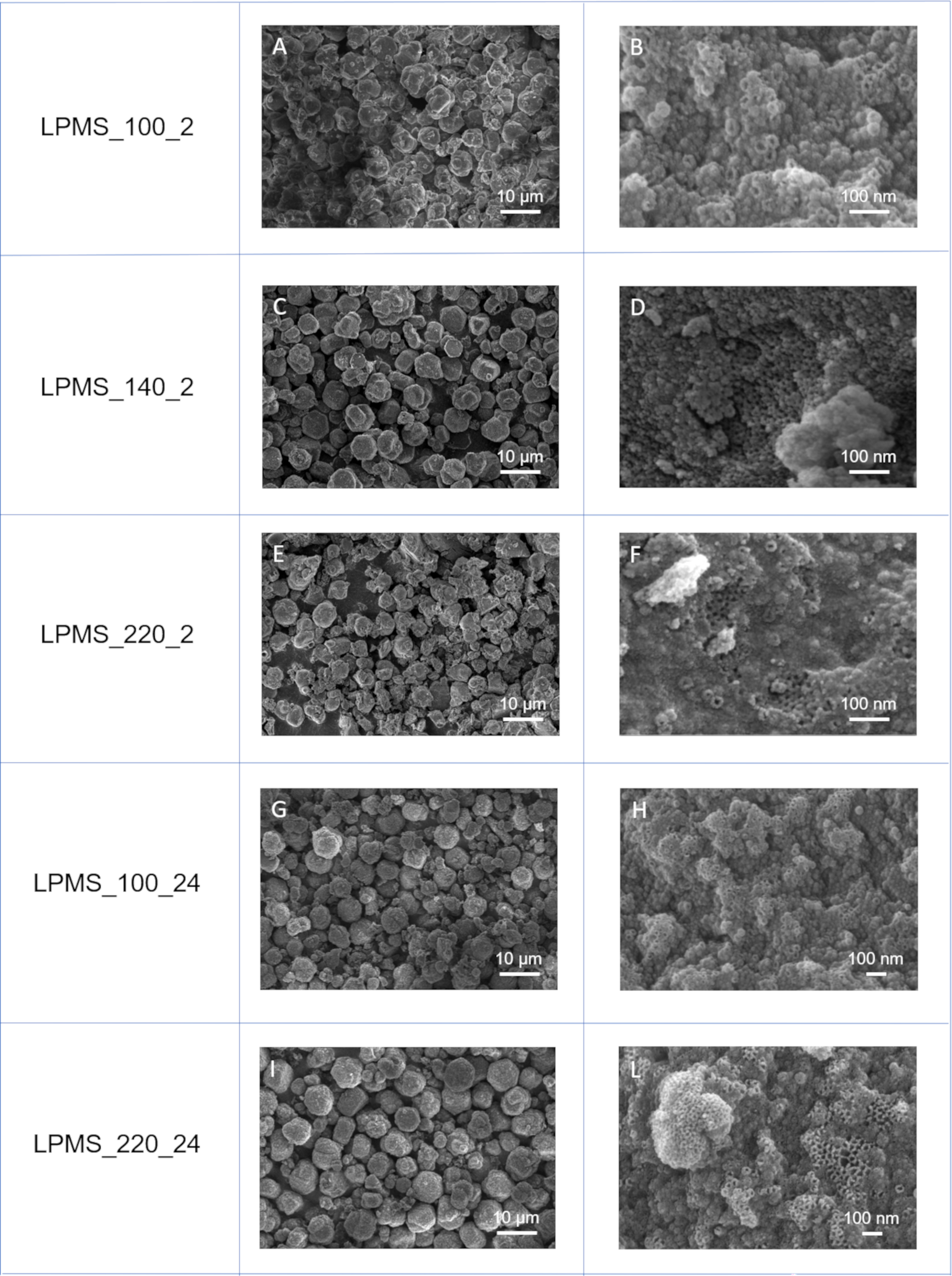

Supplement: Supplementary file 1 [file ijms-22-01718-s001.zip › SI and Figure LPMS_revised_300dpi/Figure S1.tif]

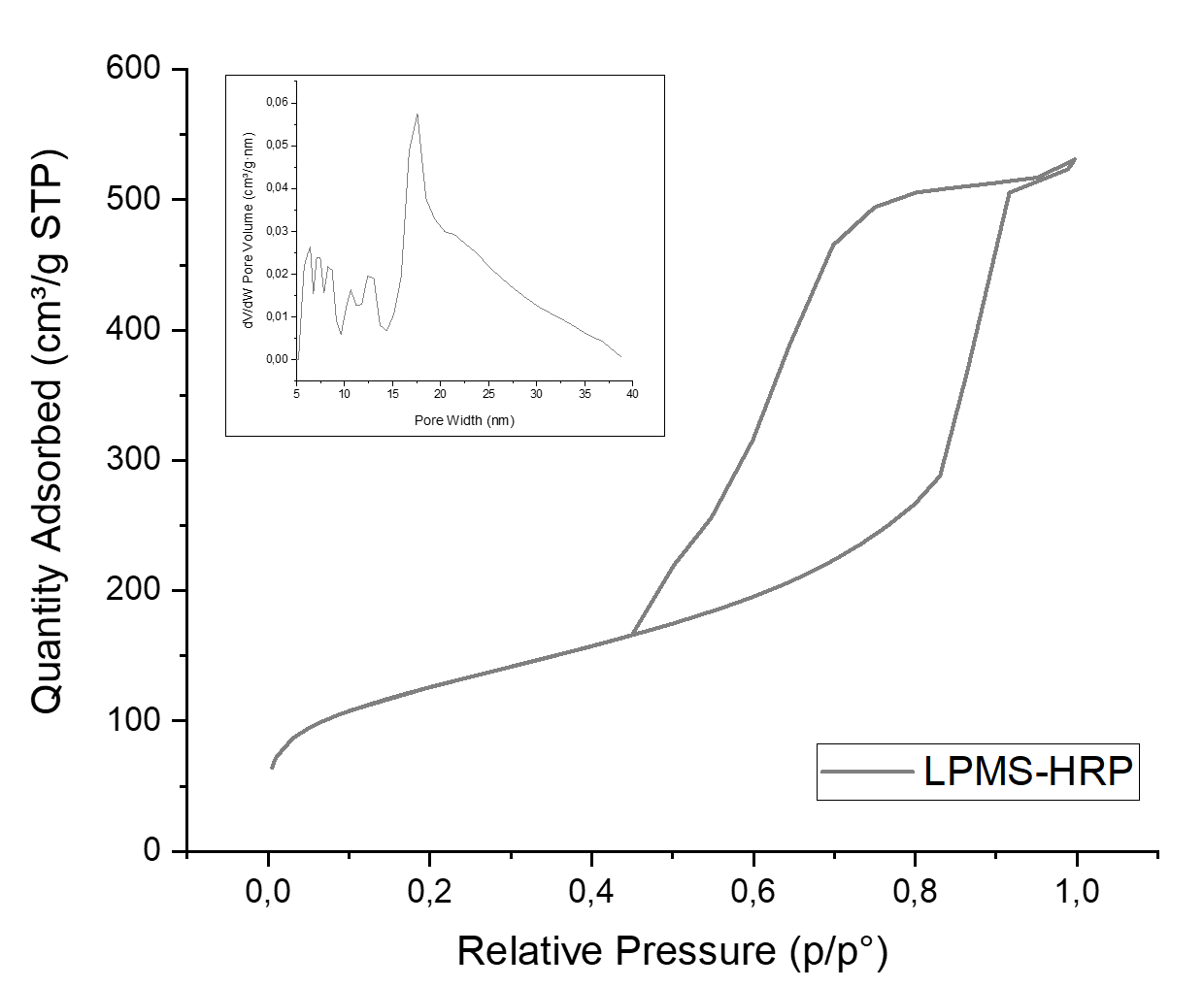

Supplement: Supplementary file 1 [file ijms-22-01718-s001.zip › SI and Figure LPMS_revised_300dpi/Figure S2.tif]

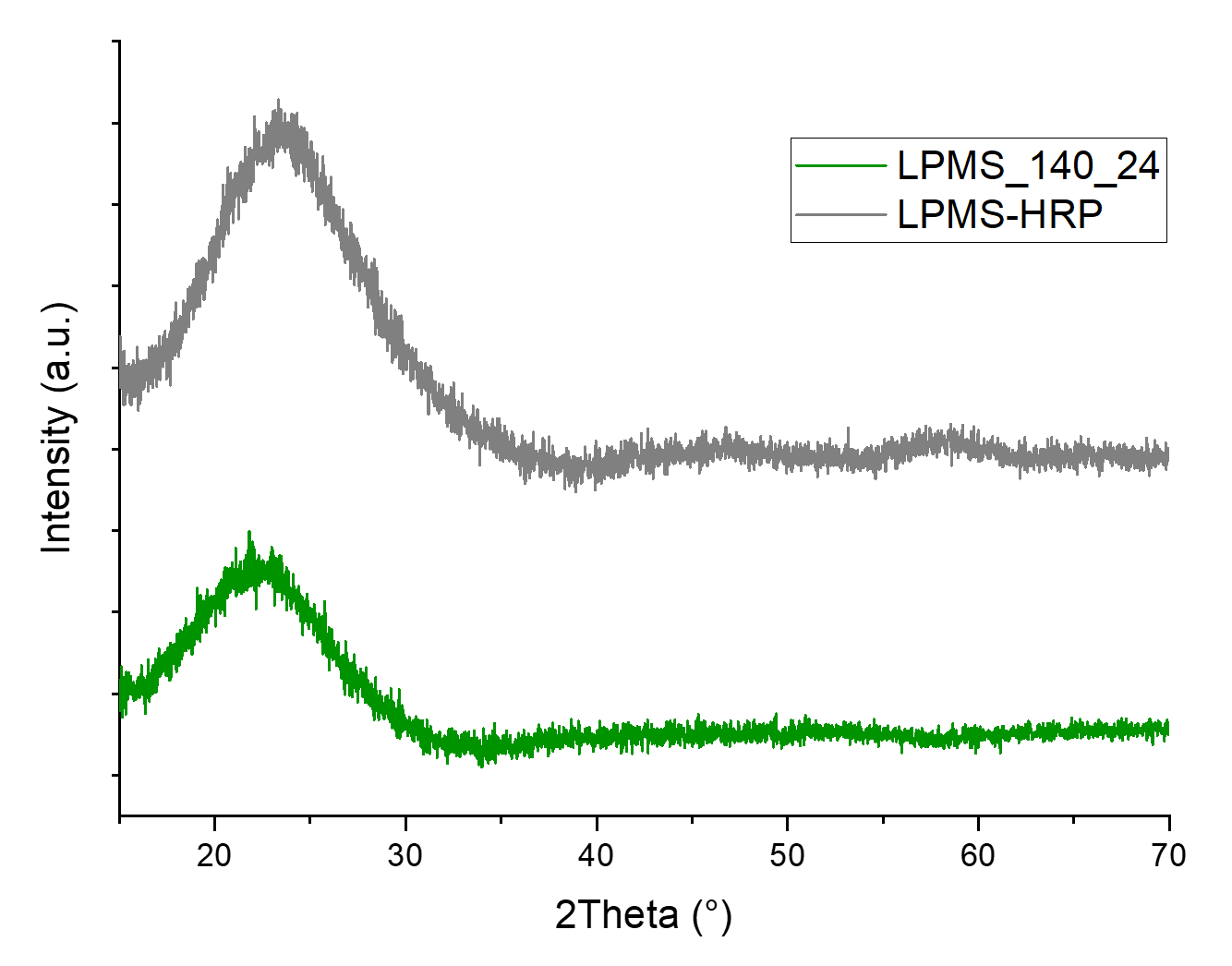

Supplement: Supplementary file 1 [file ijms-22-01718-s001.zip › SI and Figure LPMS_revised_300dpi/Figure S3.tif]

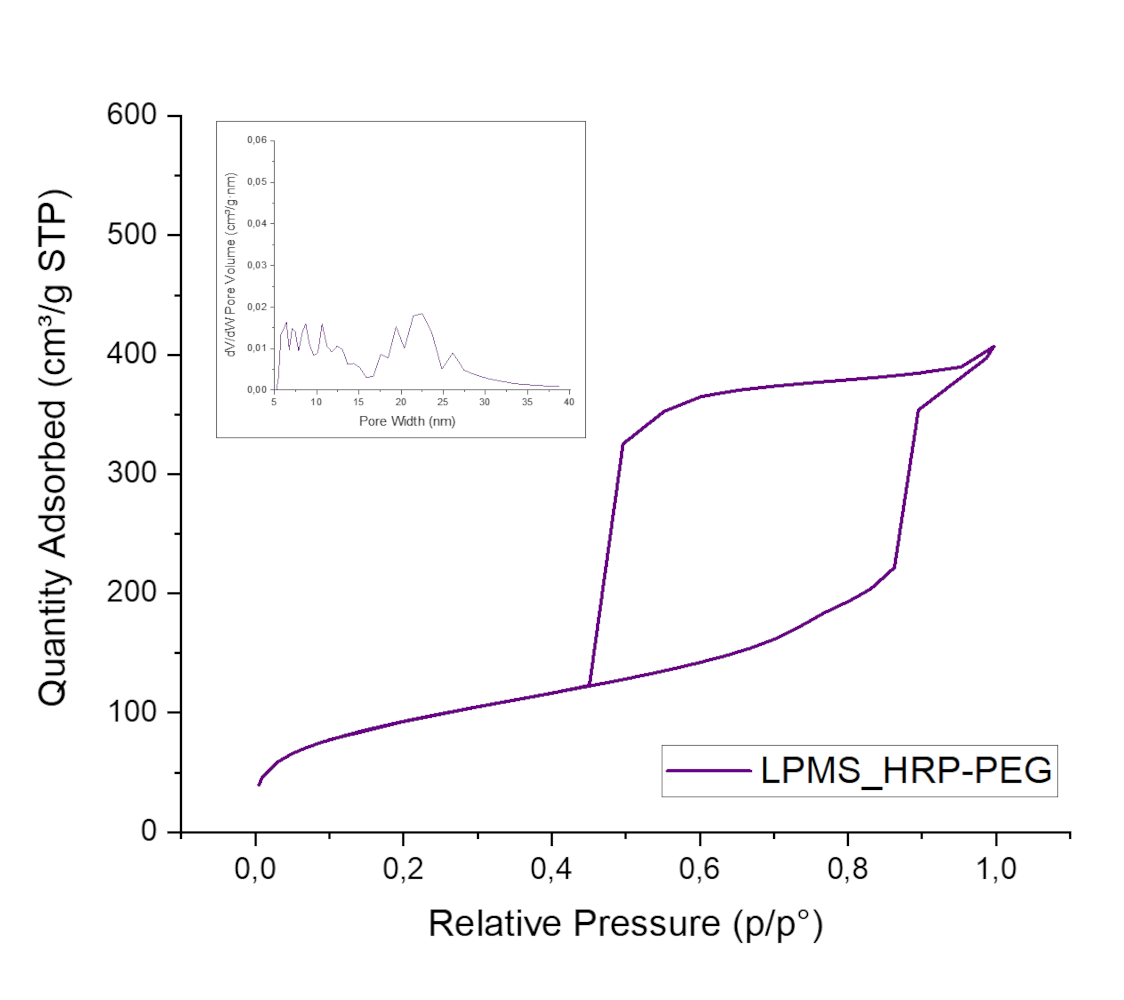

Supplement: Supplementary file 1 [file ijms-22-01718-s001.zip › SI and Figure LPMS_revised_300dpi/Figure S4_revised.tif]
